# Smart Self-Healing Capability of Asphalt Material Using Bionic Microvascular Containing Oily Rejuvenator

**DOI:** 10.3390/ma14216431

**Published:** 2021-10-27

**Authors:** Peng Yang, Li-Qing Wang, Xu Gao, Sai Wang, Jun-Feng Su

**Affiliations:** 1 School of Civil Engineering and Management, Guangzhou Maritime University, Guangzhou 510725, China; 2 School of Material Science and Engineering, Tiangong University, Tianjin 300387, China; liqingwang2021@163.com (L.-Q.W.); xugao2021@163.com (X.G.); saiwang2021@163.com (S.W.)

**Keywords:** self-healing, microvascular, asphalt, rejuvenator, microstructure, tensile test

## Abstract

It has become one of the research directions of intelligent materials for self-healing asphalt pavements to use a bionic microvascular containing oily rejuvenator. The rejuvenator in a microvascular can carry out the healing of asphalt micro-cracks, thus reducing the damage to and prolonging the life of asphalt pavement. The aim of this work was to investigate the smart self-healing capability of an asphalt/microvascular material through its microstructure and mechanical properties. Microstructure observation indicated no interface separation between the microvasculars and bitumen matrix. Micro-CT images showed that microvasculars dispersed in asphalt samples without accumulation or tangles. The phenomenon of microcracks healing without intervention was observed, which proved that the fractured asphalt sample carried out the self-healing process with the help of rejuvenator diffusing out from the broken microvasculars. The self-healing efficiency of asphalt samples was also evaluated through a tensile test considering the factors of microvasculars content, healing time and healing temperature. It was found that the tensile strength of the asphalt samples was greatly enhanced by the addition of microvasculars under a set test condition. Self-healing efficiency was enhanced with more broken microvasculars in the rupture interface of the asphalt sample. During two self-healing cycles, the self-healing efficiency of the asphalt sample with three microvascular per 1 cm^2^ of a broken interface were able to reach 80% and 86%. This proves that microvasculars containing rejuvenator play a practical role in the self-healing process of asphalt. With an increase in temperature from 0 to 30 °C, the self-healing capability of the asphalt samples increased dramatically. An increase in time increased the self-healing capability of the bitumen samples. At last, a preliminary mathematical model also deduced that the self-healing efficiency was determined by the individual healing steps, including release, penetration and diffusion of the rejuvenator agent.

## 1. Introduction

Asphalt is one of the most used pavement materials in road engineering. Microcracks will be triggered during its application because of external traffic load and external environment influence [1]. Naturally, asphalt owns a self-healing capability due to the viscoelasticity of bituminous material [2]. However, this inherent self-healing capability will be destroyed as service-time goes on. This self-healing capability has been considered as an alternative leading strategy to achieve material enhancement [3]. Nowadays, much research has been carried out to extend the service life of asphalt pavement and enhance the self-healing capability of asphalt with the assistance of smart materials [4]. These methods include polymer blending, nano modification, electrothermal conversion and rejuvenator application [5]. At present, the rejuvenator application method is considered to be an effective and simple method to enhance the self-healing capability of asphalt. As direct application has many limitations, oily rejuvenator is protected in a microcapsule or microvascular. Since 2011, Su [6,7,8,9] has systematically reported the method of microencapsulation rejuvenator for self-healing asphalt. As an intelligent engineering material, this microcapsule powder has been successfully applied in China as a commercial product [10].

Another application form of self-healing rejuvenator is to store it in a microvascular [11]. The inspiration for this idea comes from phenomena in nature, such as the self-healing of a skin injury. This concept of biomimetic microvascular self-healing was earlier applied to polymer materials [11,12,13]. Normally, bionic self-healing is carried out at the microscopic size or molecular level, and the self-healing function is achieved by the automatic connection of chemical reversible bonds or intermolecular forces. In recent years, research has indicated that this microvascular containing rejuvenator is a potentially feasible functional material for enhancing the self-healing capability of asphalt [12]. For example, Tabakovic et al. [13] prepared alginate fiber to repair asphalt pavement and found that the strength of the asphalt mortar mixture increased by 36%. Wu et al. [14] discovered that hollow calcium–alginate polymer fibers were more effective in the self-healing ability of asphalt than spaced calcium–alginate polymer fibers. Su [15] successfully fabricated a polyvinylidene fluoride (PVDF) microvascular by using a single-step spinning process. Test results proved that the microvascular dispersed in bitumen homogeneously and greatly proved the self-healing capability of bitumen. It can be imagined that asphalt using a microvascular is a promising self-healing method, which is similar to the effect of blood vessels in the human body. A cut microvascular could contain oily rejuvenator to safely resist thermal and mechanical actions [15]. Moreover, the self-healing mechanism has been investigated from the aspect of microstructure [16].

**Figure 1:**
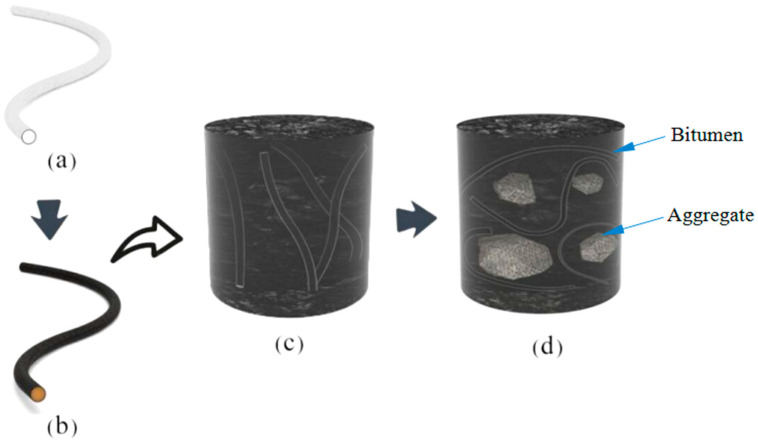
Illustration of the self-healing mechanism of asphalt using a microvascular containing oily rejuvenator, (**a**) a microvascular without rejuvenator, (**b**) a microvascular containing oily rejuvenator, (**c**) a microvascular with rejuvenator in pure bitumen, (**d**) a microvascular with rejuvenator in asphalt mixture.

It is a systematic and complex research topic to use microvascular containing rejuvenator to carry out self-healing of asphalt, which involves material preparation, structural characterization, material composite, material performance research, performance verification and practical application [15]. The entire research route can be expressed in the following simple steps, with the self-healing mechanism of asphalt using a microvascular containing oily rejuvenator as illustrated Figure 1. A hollow PVDF microvascular with a cavity structure has a white color (Figure 1a). Comparatively, Figure 1b shows a PVDF microvascular full of oily rejuvenator prepared through a spinning method. In the process of application, paraffin can be used to seal the two ends of the microvascular to avoid outflow of rejuvenator [15]. The physical and chemical properties of this microvascular have been characterized. It has been proved that this microvascular maintains good thermal stability and integrity even during the heating process of the asphalt mixture [16,17,18]. Then, the microvascular is blended into aged bitumen to investigate the self-healing capability, as shown in Figure 1c. Experimental results showed that triggered microcracks quickly disappeared and aged bitumen had a significant enhancement of self-healing capability with the help of a microvascular [17,18]. To date, the above research results have inspired us to further explore the morphology of the microvascular in asphalt and the self-healing efficiency shown in Figure 1d. Rejuvenator in the microvascular is not released in advance under the squeeze of the pebbles. When external stress acts on the microvascular, the polymeric structure of the microvascular will rupture and release rejuvenator. Oily rejuvenator molecules will penetrate and diffuse into asphalt quickly based on the power of diffusion kinetics. In total, the previous work is an initial work mainly including preparation of the microvascular, characterization of the microvascular, and observation of the morphology and integrity of the microvascular in the mixture. Based on the depth and continuity of this research, it is urgent to evaluate the self-healing capability of asphalt using a microvascular containing rejuvenator. This is a prerequisite for the basic research and practical application of this smart material.

However, the self-healing capability evaluation of asphalt has serious difficulties with characterization methods. It is well known that microcapsule or microvascular rejuvenating asphalt relies on external stress to pierce its outer shell—then the rejuvenator is released to complete the self-healing process [19]. There is no perfect observation and characterization method for evaluating self-healing efficiency. For asphalt material, the commonly used traditional method to detect the self-healing efficiency is primarily the three-point bending (3PB) method, which is a very convincing experiment. Su et al. [20] studied and improved a 3PB test system to evaluate asphalt mixtures containing microcapsules with rejuvenator. Researchers [21] also used the above system to evaluate the self-healing effect of mortar mixtures. It is disappointing that the 3PB experiment can only provide relatively macroscopic evidence for self-healing efficiency. This approach may be not applicable for observing this self-healing asphalt using a microvascular. One reason is that there is no evidence that the microvasculars can be broken in the asphalt through compression elastic deformation in the 3PB method. The specific direction of stress extension is unknown and aged asphalt can develop stress everywhere. Another reason is that the polymeric microvasculars are difficult to break under pressure. At the same time, it is necessary to study the general development of microcracks and observe the microcracks under the action of the healing agent, and there are few reports on the research of self-healing capabilities from a micro perspective. In addition, tensile fracture behavior is suitable for characterizing mechanical behavior and self-healing due to the tensile elongation of the microvascular.

Based on the above analysis, the aim of this paper was to investigate the self-healing capability of asphalt material using a microvascular containing oily rejuvenator through microstructure analysis and mechanical property investigation. In this way, self-healing phenomena were observed directly using several morphology characteristic tools. The observed evidence opens up the research of microcrack self-healing in asphalt with microvascular, which fills a gap in this research field. Following this, the self-healing efficiency capability was evaluated by comparing tensile breaking strength before and after one/two self-healing cycles. The self-healing efficiency was measured by the recovery degree of tensile fracture strength. In sum, microstructure observation and macroscopic mechanical properties will both supply more convincing evidence of the self-healing capability of asphalt. There is no doubt that the systematic consideration of microstructure and mechanical properties can provide more references for the self-healing behaviors of asphalt using microvascular containing rejuvenating.

## 2. Experimental

### 2.1. Materials

The bitumen (80/100 penetration grade), provided by Qilu Petrochemical Industries Co. (Zibo, Shandong Province, China), was manually processed through a thin film oven test into the aged bitumen (40/50 penetration grade). Pebble was leftovers from road construction. Polyvinylidene fluoride (PVDF, 6010#) and N, N-Dimenthylacetimide (DMAc, 98%) were both purchased from Solvay Advanced Polymers (Parachute, Co., Dongguan, Guangdong, China). Oily rejuvenator was supplied by Tianjin Sinogo Co. Ltd. (0.905g/cm^3^, 4.240 Pa·s, Tianjin, China).

### 2.2. Preparation of Microvascular Containing Rejuvenator

Microvascular containing rejuvenator was fabricated by dry–wet method spinning. In view of the previous detailed work [15], a brief introduction is as follows: The PVDF powder was first dried in an electric oven at 60 °C for 4 h, then PVDF (100 g) and DMAc (400 g) were heated and stirred at 60 °C for 8 h in a sealed three-neck round bottom flask to prepare a uniform pouring liquid. The casting solution was put at vacuum pressure for 24 h to eliminate bubbles. Then it was poured into the spinning storage tank at a constant temperature of 60 °C and the spinneret started to work under the pressure of nitrogen gas (N_2_, 0.15 MPa). At the same time, the oil rejuvenator liquid flowed into the core cavity, which was powered by a pressure of 0.2 MPa. The casting solution and core liquid were both injected into the spinneret and microvasculars containing rejuvenator were formed. Meanwhile, the temperature of the coagulation bath was maintained at 25 °C. The microvascular was wound around the winding wheel powered by a guide wheel, and the ends of microvascular were sealed by a heat sealer. Finally, the microvascular was soaked in distilled water at room temperature for later use.

### 2.3. Mixture of Microvascular in Asphalt

The long microvascular was uniformly cut into 2.0 cm segments and each end was sealed with paraffin. The short ones and the pebble were randomly mixed in asphalt with a cylindrical silicone mold. The samples were put in a refrigerator at −10 °C for 24 h and used for micro-CT scanning. In addition, a square sample was prepared in the same way to observe the self-repair process. The difference is that the sample contained a small amount of aggregate. The square sample was placed under a low temperature of 0 °C for 2 days, and then liquid nitrogen was dropped in the middle of the sample to make it brittle and fractured. Then, the broken two parts were spliced together and placed under 0 °C in the same environment as the original sample for 3 days.

### 2.4. Morphology Analysis

The microvasculars and molten asphalt were randomly mixed to make a sample, which was placed at 0 °C for 2 days, and then sliced. The surface morphology and integrity of the microvascular as well as the state of the mixed in bitumen were monitored by a scanning electron microscopy (SEM, Nanosem-430, FEI, Hillsboro, OR, USA).

### 2.5. Micro-CT Obversion

Micro X-ray computed tomography (Micro-XCT 400, Xradia, NY, USA) was applied to observe the distribution of self-healing microvascular in bituminous binder. The sample was carefully installed on the holder and was guaranteed to be within a reasonable radiation range. X-rays were emitted from the radioactive source and penetrated the sample. Furthermore, the two-dimensional images were reconstructed to 3D-modeling by built-in 3D modeling software.

### 2.6. Observation of the Rejuvenator Diffusion during a Self-Healing Process

A glass slide was taken and coated with a thick layer of asphalt. The asphalt was covered with a microvascular of the same length as the glass slide. The sample was put in a refrigerator at 0 °C for 24 h, and then liquid nitrogen added to it to trigger the microcracks. The state of the rejuvenator and the diffusion behavior were both observed by a fluorescence microscope (FR-4A Cossim, Beijing, China).

### 2.7. Investigation of the Self-Healing Capability by Mechanical Tests

**Figure 2:**
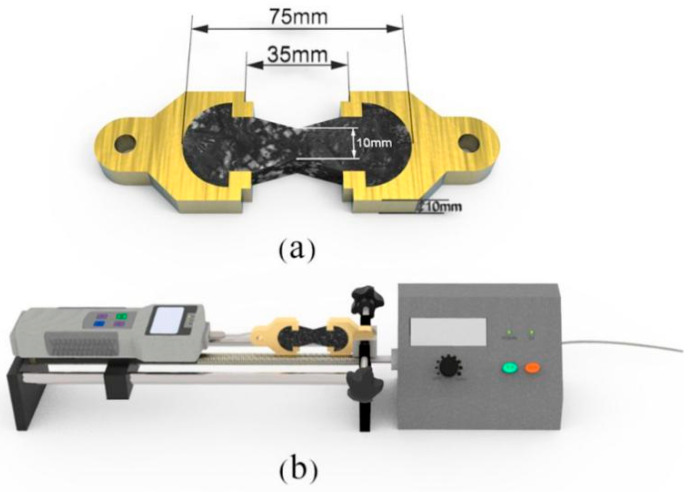
Illustration of the tensile property testing mold, (**a**) testing mold and (**b**) machine model.

**Figure 3:**
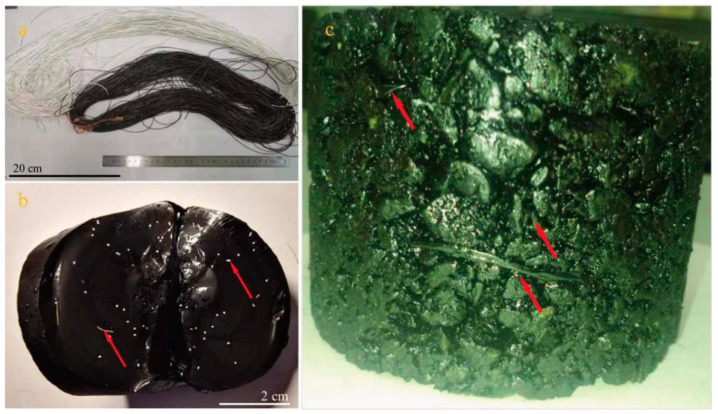
Photographs of microvasculars in asphalt sample, (**a**) microvascular without (white) or with (black) oily rejuvenator, (**b**) interface morphology of broken bitumen sample with microvasculars without oily rejuvenator, and (**c**) asphalt sample with microvasculars with rejuvenator.

The self-healing capability was tested by a tensile machine with a sensor (±2.5% RH, ±1 °C, 0.01N; Aigu, NK-ZP 10-1000 N, Fuma Electric Equipment Co., Shenzhen, China). Figure 2 illustrates the tensile model and tensile machine including the shape and size. This geometry ensures the stretching direction is in the center of the sample. The microvasculars were cut into equal lengths of 3 cm and terminated with paraffin wax (Figure 3a). Aggregate, microvascular segments, and molten asphalt mixture were poured into a copper mold, left to cool to normal temperature, and put in a refrigerator at −10 °C for 72 h. The frozen samples were subjected to a tensile test at room temperature (Figure 3b). The drawing speed could be controlled by the rotation speed of the crank. The tensile breaking strength was directly recorded by the sensor noted as (*T_b_*_0_). After being broken, each sample was placed in an environment of −3 °C for 5 days according to the shape of the original samples. Its tensile strength was measured again, recorded as (*T_b_*_1_). Then, the healed sample can be stretched to fracture with a tensile breaking strength (*T_b_*_2_) and self-healing for the second cycle. The self-healing capability is calculated according to the ratio of two values as Equations (1) and (2) show:

(1) $${\mathrm{SHC}}_{1}=\frac{T_{b1}}{T_{b0}}\times 100\%$$

(2) $${\mathrm{SHC}}_{2}=\frac{T_{b2}}{T_{b0}}\times 100\%$$

where SHC_1_ is the self-healing capability of sample in the first cycle, SHC_2_ is the self-healing capability of sample in the second cycle, *T_b_*_0_ is the original tensile breaking strength, *T_b_*_1_ is the tensile breaking strength of the asphalt sample after the first self-healing process, and *T_b_*_2_ is the tensile breaking strength of the asphalt sample after the second self-healing process.

## 3. Results and Discussion

### 3.1. Observing Interface Microstructure of Microvasculars in Asphalt Sample

Interface microstructure is essential in composite material research [22]. The stability of the interface structure can ensure the stability of the composite material. Therefore, it is necessary to observe the interface of the microstructure in the asphalt/microvascular sample in this work. Interface microstructure observation is more conducive to a deeper exploration of the connection interface of a composite material. Firstly, the macroscopic appearance of an asphalt/microvascular sample is observed to provide a general understanding of the basic structure of the material. Figure 3a shows microvasculars without (white) or with (black) oily rejuvenator. Figure 3b shows the interface morphology of broken bitumen sample with microvasculars without oily rejuvenator. The microvascular without rejuvenator is hollow and its color is white.

Part of the interface junction is marked with a red arrow. Figure 3c shows a photograph of an asphalt sample with microvasculars. Red arrows mark the embedded microvasculars in the mixture of aggregate and bitumen. The preparation of this sample takes into account the horizontal and vertical distribution of the microvasculars, and also provides us with ideas for studying the dispersion of the rejuvenator in different directions.

**Figure 4:**
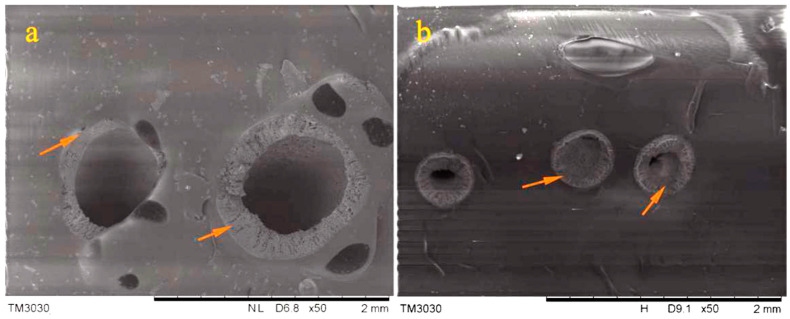
SEM interface morphology of microvasculars in asphalt sample, (**a**) hollow microvasculars without oily rejuvenator tightly connected with the asphalt material, (**b**) interface morphology of the microvasculars containing rejuvenator in the asphalt.

The stability of the interface connection plays a decisive role in the performance of the composite material [23]. The interface microstructure should be studied and the debonding phenomenon should be avoided. Additionally, the debonding will block the propagation of microcracks and hinder the initiation of microvascular rupture. Figure 4 presents the SEM interface morphology of microvasculars in the asphalt sample. As shown in Figure 4a, it can be clearly seen that the hollow microvascular without oily rejuvenator is tightly connected with the asphalt material, and there is no shedding. Figure 4b provides the interface morphology of the microvasculars containing rejuvenator in the asphalt. There is no interface separation between microvasculars and the bitumen matrix, which has also been confirmed in previous work [16]. In addition, oily rejuvenator also completely fills the entire microvascular, increasing the amount of rejuvenator–which will improve the healing effect.

### 3.2. Distribution States of the Microvascular in an Asphalt Sample

It is an important issue that the distribution of the microvascular in asphalt should be even, without entanglement. In order to investigate the distribution of a microvascular in asphalt, the asphalt samples were scanned by micro-XCT, which provided an in-situ observation. Micro-XCT scans the material with X-rays to obtain the internal structure of the material, whose principle comes from the fact that different densities of the material have different gray levels [24].

**Figure 5:**
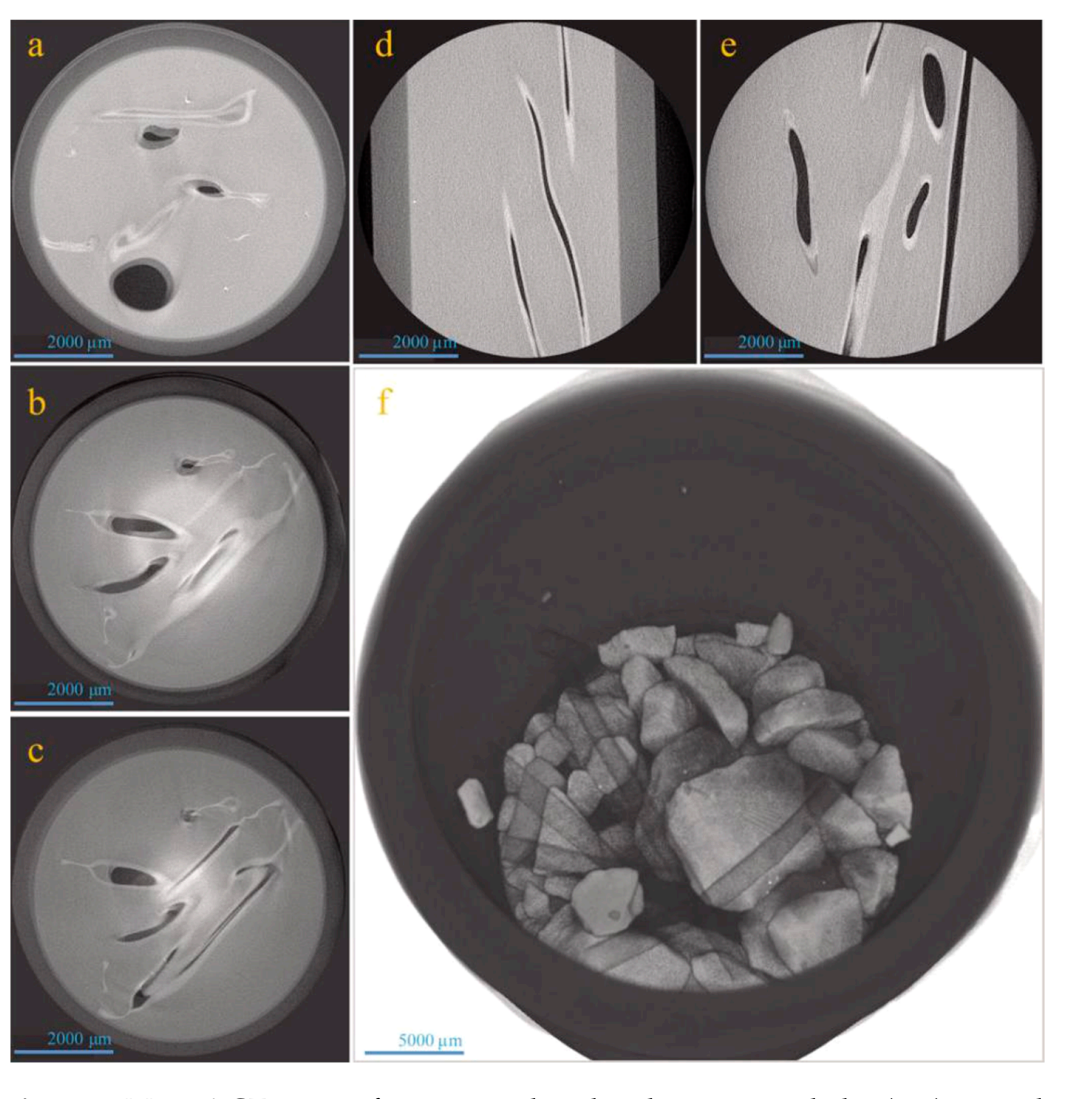
Micro-XCT image of microvasculars distribution in asphalts, (**a**–**c**) vertical view images of an asphalt sample with microvasculars, (**d**,**e**) side view images of an asphalt sample with microvasculars, and (**f**) 3D image of an asphalt sample with microvasculars.

Figure 5 displays several micro-XCT images of multi-microvasculars distribution in asphalt samples. Figure 5a–c shows vertical-view micro-XCT slides of an asphalt sample. It can be seen that some microvasculars stand upright in the cylindrical sample, and some microvasculars are placed horizontally under the squeeze of the aggregate. Side-view micro-XCT slides of an asphalt sample with microvasculars are shown in Figure 5d,e. It can be found that no entanglement appears between the microvasculars. Figure 5f shows a 3D micro-XCT image of asphalt sample with microvasculars, which is synthesized by its own modeling software. The aggregates and microvascular can clearly be distinguished in this image. The larger density makes the aggregate very clear, and the smaller density microvasculars can only show a black transparent tube shape. The distribution of microvasculars is still clearly visible. Microvasculars are evenly dispersed without any entanglement, which is consistent to the vertical-view and side-view results. At the same time, the microvasculars keep perfect integrity without any damage.

### 3.3. Observing the Microcrack Self-Healing Process

The self-healing function of asphalt is inherent feature of its own. This capability gradually declines with time, environment and application [25]. The appearance of cracks is due to the existence of external stress and piercing of the aged asphalt pavement. In order to observe the rupture of microvasculars, artificial cracks were stimulated to study the self-healing process in this work.

**Figure 6:**
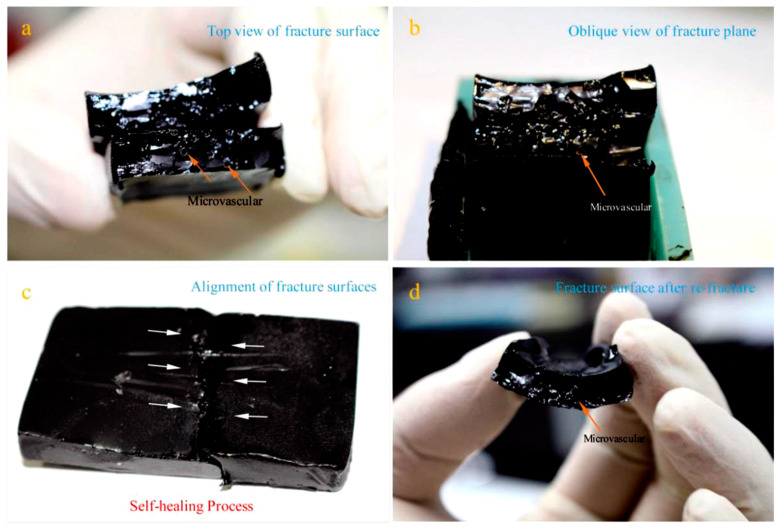
Photographs of the self-healing process of an asphalt/microvascular sample, (**a**) top view of the fracture surface of the asphalt sample, (**b**) oblique view of the fracture surface of the asphalt sample, (**c**) alignment of the fracture surface of the asphalt sample with a self-healing process for 3 days, and (**d**) the fracture surface of the re-fracture.

Figure 6a,b shows the top-view and oblique-view of the fracture surface of asphalt samples, and Figure 6b shows the oblique view of the fracture surface of asphalt samples with broken microvasculars under 0 °C, as the arrows point out. It can be clearly seen that the asphalt fracture surface is relatively rough and not smooth due to the presence of microvasculars. Following this, the self-healing process was carried out slowly with the help of the rejuvenator. Figure 6c shows the alignment of fracture surfaces of the asphalt sample. Three days later, the sample was broken again—Figure 6d shows the photograph of the fracture surface of re-fracture. Oily rejuvenator in microvasculars was consumed with a hollow structure.

### 3.4. Rejuvenator Diffusion during a Self-Healing Process

**Figure 7:**
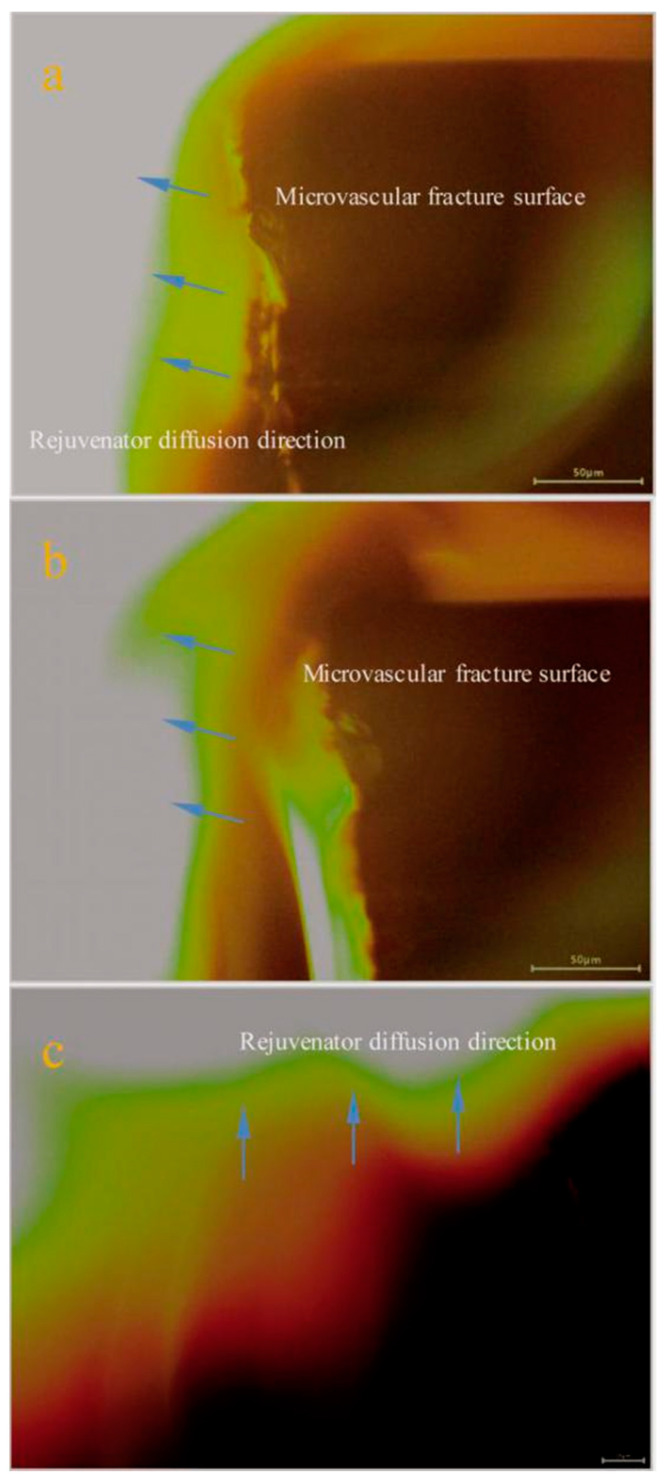
Fluorescence micrography of rejuvenator diffusion into asphalt sample during a self-healing process, (**a**) oily rejuvenator diffusion into asphalt from a fracture microvascular, (**b**,**c**) oily rejuvenator diffusion rapidly into an asphalt sample during 2 h and 4 h under 0 °C.

It has been reported that a self-healing process in the asphalt/microvascular system is a periodic continuous movement of liquid rejuvenator, including its steps of penetration, release, capillary, and diffusion [16]. Diffusion is an important aspect of the self-healing process, which leads to rejuvenator molecules spreading to a larger extent on the fracture surface. To measure the diffusion behaviors, it is essential to understand the microvascular states. One microvascular containing rejuvenator was mixed into an asphalt sample; the bitumen had a 40/50 penetration grade. Liquid N_2_ was dripped on the top of this asphalt sample to trigger a rupture. The cross-section of the broken microvascular could be clearly observed on the fracture surface of this asphalt. The rejuvenator diffusion details were directly identified using a fluorescence microscope under 0 °C. The diffusion rate is strongly dependent on temperature, because bitumen is a temperature-sensitive material. Therefore, it is easy to observe the diffusion behavior at a low temperature when the temperature is fixed at zero. Interestingly, the green color of rejuvenator in fluorescence microscope images can indicate the diffusion range [15]. In Figure 7a, oily rejuvenator is observed flowing out from one end of a broken microvascular. After 2 h even under a low temperature, it can be identified that the diffusion area of rejuvenator is expanded about 50 μm (Figure 7b). The arrows point in the direction of the diffusion. From the perspective of diffusion dynamics, rejuvenator molecules have a diffusion capability based on the motive force of a concentration gradient. It means that the molecules have a tendency to move from a higher concentration to a lower concentration. After 4 h under 0 °C, the rejuvenator has an even wider diffusion range (Figure 7c). From the region and shade of green color around the microvascular, the diffusion area can be measured directly. A wavy diffusion area is attributed to the various speed of rejuvenator at different points. Due to the above diffusion phenomena, small molecules of rejuvenator can dramatically soften the asphalt material and practically recover its original performance, especially its self-healing capability.

### 3.5. Design the Self-Healing Test Method

The above tests give some actual results on the self-healing capability, reflecting some basic rules. However, they cannot really predict the self-healing efficiency of asphalt under certain environments and conditions. Of course, the mathematical model can better reflect the relationship between the influencing factors and the self-healing efficiency, which is helpful in the design of the material structure and the prediction of self-healing efficiency in practical application. The continuous revision of the mathematical model will guide the theory of material structure design and help to improve the self-healing efficiency at the same time. In this work, it is necessary to design the microstructure and asphalt and the self-healing test method before the self-healing efficiency evaluation. Simplification of a complex material and the giving of boundary conditions of the test will help to solve the self-healing problem.

It is one of the most common methods to measure the self-healing capability of materials by tensile fracture experiments [26]. The self-healing efficiency can be calculated based on a repetitive tensile force through microcrack generation and crack healing during various rest periods [27]. Microcracking is one of the main distresses that are responsible for the service-life reduction of asphalt pavement. Therefore, an understanding of crack healing behavior is important for service-life prediction. The fracture–healing–refracture tests will help to investigate self-healing capability during a loading–healing–reloading process. For example, Qiu [26] has reported that self-healing behaviors are dependent on the healing time, temperature, crack phase, material modifications and bitumen aging degree. Su [28] also designed a repetitive tensile method to measure the self-healing behaviors of bitumen samples by comparing the self-healing efficiency at various rest periods and temperatures. It was found that the content and orientation of microvasculars in the bitumen influenced the self-healing behavior. At the same time, an increase in temperature and time enhanced the self-healing efficiency according to the time–temperature superposition principle. Because of its visco-elastic nature, a self-healing behavior is considered to be a process of crack closure and strength gain with the help of a visco-elastic feature under a certain temperature for a period of time [29].

**Figure 8:**
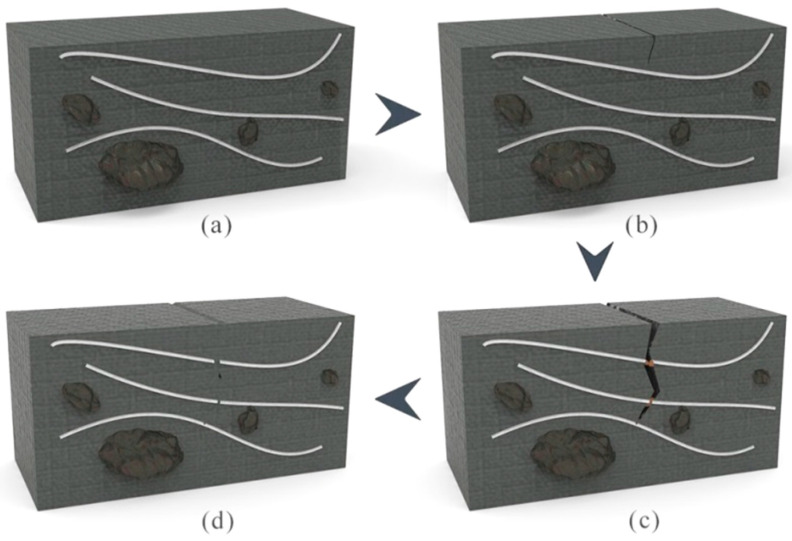
Illustration of the process of microvasculars rejuvenating asphalt, (**a**) an asphalt sample with multi-microvasculars, (**b**) microcrack triggered, (**c**) tip-stress of a microcrack pierced the microvasculars, and (**d**) rejuvenator in microvasculars released out.

Microstructures of asphalt material are more complex, consisting of bitumen and aggregate. In order to reflect the details of the microstructure, an illustration is used to express the mechanism of the self-healing process of the asphalt/microvascular composite, as shown in Figure 8. Firstly, Figure 8a displays the initial state of microvasculars input into the asphalt. The sample is composited of bitumen, aggregate and microvascular. The microvasculars are basically in a parallel state, there is no cross-connection and entanglement. In Figure 8b, a microcrack is triggered on top of this sample. The continuous expansion of the microcracks will cause the fracture of the microvasculars (Figure 8c). The strength of the tip-stress of the microcrack determines the number of the punctured microvasculars and the speed of rupture of the microvascular. Immediately, oily rejuvenator releases out of broken microvasculars under the force of capillarity. Under the action of the concentration difference, the oily rejuvenator rapidly penetrates and diffuses into the bituminous material around the microcrack. The bitumen on both sides of the microcrack will be softened and its viscosity increased. Under proper temperature conditions and with sufficient time, the microcrack will heal gradually (Figure 8d). Obviously, the release and diffusion rates of the rejuvenator will determine the rate of the self-healing process.

Based on the above analysis of the self-healing mechanism of asphalt, it can be imagined that the mechanical properties of asphalt materials will be definitely affected by different components and different aggregate shapes. When the microvascular material is added in asphalt, its structure is even more complex. Furthermore, the influence factors of mechanical properties will be more and more difficult to control due to the superposition effect. Besides the complexity of the material structure, the distribution direction of microvasculars also greatly affects the performance of the asphalt. Previous work found that only a slight decrease in self-healing efficiency during the first and second self-healing cycles with a microvascular orientation between 15°–45° [17]. The orientation is the angle between the tensile direction and the microvascular. This phenomenon means that the microvascular orientation nearly does not influence the healing behaviors during the original two healing cycles. At the initiation of healing, the rejuvenator will exhibit nearly the same diffusion behaviors. Meanwhile, a larger microvascular orientation will block the flow of oily rejuvenator. The rejuvenator may have less opportunity to diffuse into the rupture interface of the bitumen sample. Therefore, in order to provide more convenient self-healing efficiency, the structure of the above asphalt materials is simplified with a microvascular orientation between 0°. This method has also successfully been applied in previous work [17]. In other words, the direction of the microvasculars is parallel to the direction of the tensile. On another hand, the microvascular contents will also greatly influence the self-healing efficiency of asphalt [15]. An increase of microvascular contents on the rupture interface of the microcrack allows more rejuvenator molecules to diffuse into the asphalt. This mechanism has also been found in previous work reporting that multi-microvasculars influenced bituminous material self-healing behavior significantly [15]. Therefore, in order to provide a more convenient self-healing evaluation, the tension test of this work was simplified with the following conditions:

- The orientation of the microvasculars in asphalt was roughly in the same direction of the tensile.
- The number of microvasculars was 1–3 in the rupture interface of the asphalt testing samples (1 cm^2^).
- The test temperature was 0 °C.
- A self-healing cycle was 24 h. Two cycles were carried out for each asphalt sample in this work.

### 3.6. Self-Healing Efficiency Influenced by the Microvascular Contents

**Figure 9:**
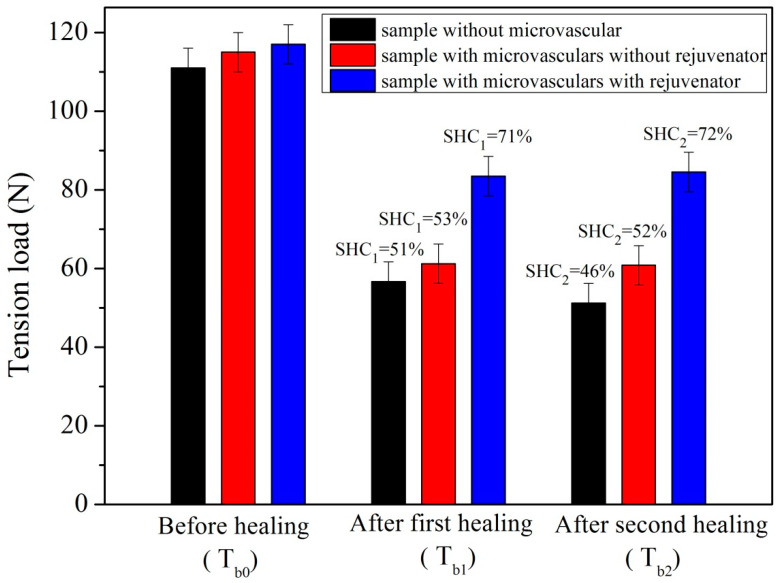
Tension load values and self-healing capability (SHC_1_ and SHC_2_) values of three asphalt samples (one sample without microvascular, one sample with microvascular without rejuvenator, and one sample with microvascular containing rejuvenator) under 0 °C during two self-healing cycles, each self-healing cycle carried out for 24 h.

Figure 9 shows tensile load values and SHC values (SHC_1_ and SHC_2_) of three asphalt samples influenced by microvascular contents (1–3) under 0 °C during two self-healing cycles. They are one sample without microvascular, one sample with microvascular without rejuvenator, and one sample with microvascular containing rejuvenator, respectively. Each self-healing cycle was carried out for 24 h. By horizontally comparing the self-healing case of the three samples under the same conditions, we can further demonstrate the actual action of the microvascular presence. Before a self-healing process, the tension load value of the asphalt sample without the microvascular has the minimum tension load value, which is due to the microvascular characteristics of strengthening and toughening for asphalt material [30]. Comparing reinforcement characteristics of microvasculars containing rejuvenator and microvasculars without rejuvenator on asphalt materials, the original tension load values of the two samples were almost equal. After the first cycle self-healing cycle, three samples had SHC_1_ values of 51%, 53% and 71%. It can thus be inferred that regenerants play a decisive role in terms of the self-healing process. The fractured samples were placed under 0 °C according to the original appearance of the samples, and allowed to undergo the second self-healing cycle for 24 h. It can be identified that two samples without rejuvenator had a decrease in SHC values. Meanwhile, the sample with rejuvenator had an even higher SHC_2_ value of 72% compared to the other two samples (46% and 52%). The SHC_2_ value of the sample without the microvascular was slightly lower than its first self-healing cycle. The possible reason was attributed to the absence of microvascular reinforcement and toughness. The asphalt material underwent a fracture–healing–refracture experiment with serious aging. The bituminous molecules were untangled and the intermolecular forces were reduced. Its self-healing ability was destroyed with the extension of service time. Simultaneously, it can be indicated that the rejuvenator improved the self-healing capability of the asphalt compared to the tension load values of asphalt/microvascular samples with/without rejuvenator. Rejuvenator does have a key role in the self-healing process.

**Figure 10:**
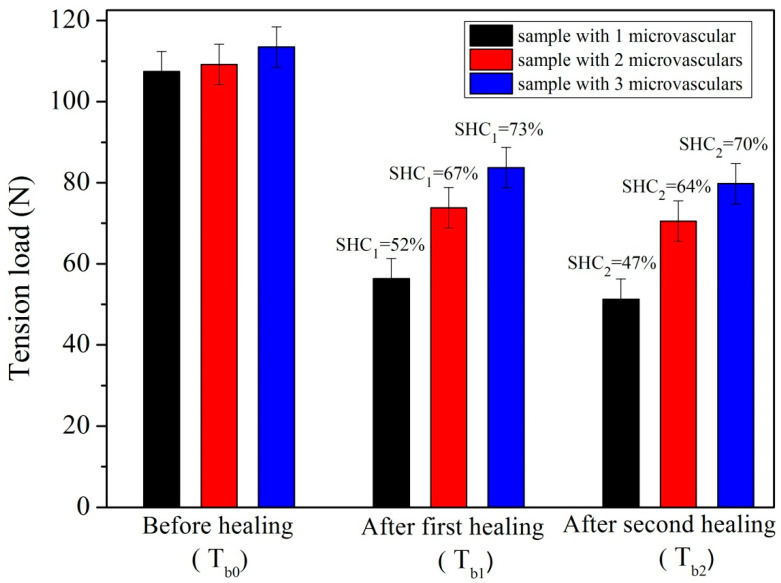
Tension load values and self-healing efficiency (SHC_1_ and SHC_2_) values of asphalt samples influenced by microvascular contents (1–3, paralleling to tension direction) under 0 °C during two self-healing cycles.

Figure 10 shows the tension load value and self-healing efficiency (SHC_1_ and SHC_2_) of asphalt samples with different microvascular contents (1–3 microvasculars) at 0 °C during two self-healing cycles. All microvasculars in the asphalt matrix were parallel to the tension direction. Asphalt and the microvascular were composed of composite materials, which generally have an enhanced strength of asphalt [31]. The tension load values of the original samples were approximately 108, 111 and 115 N, which fully reflects that the number of microvasculars affects the tension strength of the asphalt material to a certain extent. At a low temperature, the fracture of the asphalt sample may have a brittle rupture with a lower fiber impact. After the first self-healing of 24 h, the three samples had the tension load value of 58 N, 76 N and 82 N. Meanwhile, the three samples had the tension load value of 44 N, 65 N and 78 N after the second self-healing of 24 h. Three samples had the SHC_1_ values of 52%, 67% and 73%, respectively. Firstly, it is found that the tension load was decreased for each sample. The existence of a self-healing microvascular cannot recover the mechanical properties to their original state. This conclusion has also been drawn in the microencapsulated rejuvenator applied in bitumen [7]. When the strength is lower than a certain limit point, the aged bitumen can no longer recover its properties and the self-healing capability has a maximum threshold. It has also been found that asphalt needs more time to recover its properties with more self-healing cycles. Secondly, these data indicate that the existence of microvasculars really enhances the recovery of mechanical properties and improves the self-healing capability of the asphalt samples. Similar results were found in their SHC_2_ value (47%, 64% and 70%) in the second self-healing cycle. For each sample, its SHC_2_ value was dramatically less than the SHC_1_. This phenomenon means that the multi-self-healing efficiency will be reduced significantly, because the rejuvenator may exhibit the largest diffusion behavior in the first self-healing cycle [20].

### 3.7. Self-Healing Efficiency Influenced by Time and Temperature

**Figure 11:**
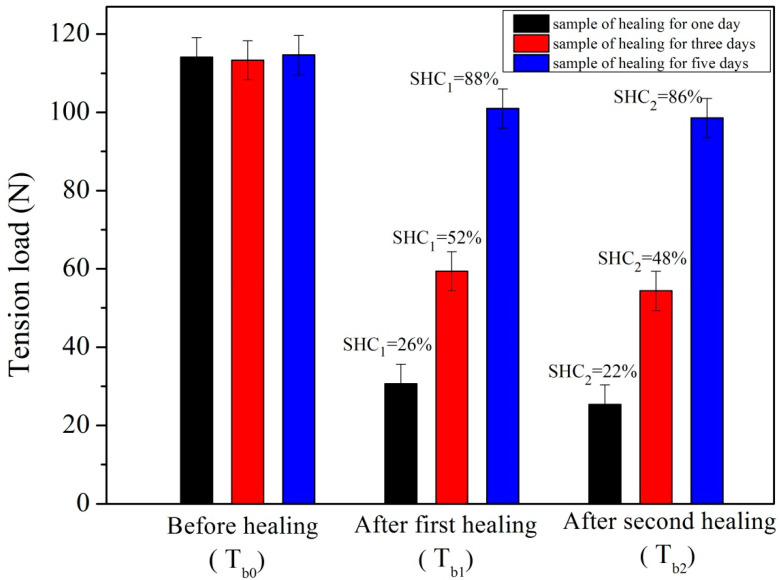
Tension load values and self-healing efficiency (SHC_1_ and SHC_2_) values of asphalt samples with 3 microvasculars influenced by time in two self-healing cycles under 0 °C, each self-healing carried out for 1, 3, and 5 days.

Time is a factor influencing the self-healing properties of asphalt because visco-elastic molecules can regulate its status with sufficient time. In other words, bituminous material has sufficient time to wet and entangle molecular chains [32]. Rejuvenator molecules need a relative longer time to penetrate and diffuse into aged bitumen and recover the original character of bitumen [33]. In this work, a simplified experimental process was designed to identify the influence of time. Figure 11 shows the tension load values and self-healing efficiency (SHC) values of asphalt samples with three microvasculars during two self-healing cycles at 0 °C. Each self-healing cycle has a healing-time of one, three and five days, respectively. The reason for the setting of five days as a maximum time is that previous work has proved that pure bitumen can complete a self-healing process under this condition easily [17]. Three asphalt samples have a similar original tension load of 114 N. After the first self-healing process of 1, 3 and 5 days, they had the tension load values of 30, 57 and 101 N. By calculation, their SHC_1_ values were 26%, 52% and 88% respectively. After the second self-healing process of 1, 3 and 5 days, their SHC_2_ values were 22%, 48% and 86%. According to the data obtained from the above experiments, two conclusions can be drawn. Firstly, SHC of asphalt decreases with increases in self-healing cycles. The reason for this is that the self-healing capability of asphalt has a gradual decay characteristic. This conclusion is basically consistent with the previous analysis [17]. Secondly, the SHC of the asphalt sample increases with more time over the same self-healing cycle. In previous work, it has been found that the SHC value of aged bitumen (40/50) samples were all similar, at nearly about 50% at the beginning of the two days [17]. This value decreased to 45% over the next four days. Without the external assistance of rejuvenator, aged bitumen does not provide an enhanced self-healing capability [16,33,34]. In this work, asphalt samples can nearly recover to more than 80% of their original tension load value after two self-healing cycles over 10 days. Therefore, it can be identified that time can enhance the self-healing capability of asphalt significantly. However, the SHC of the same sample does not increase in the next self-healing process if the time is extended in each cycle. That is to say, the indefinite extension of time cannot enhance the SHC value at the same time. Each asphalt sample has a threshold of time during a self-healing process.

**Figure 12:**
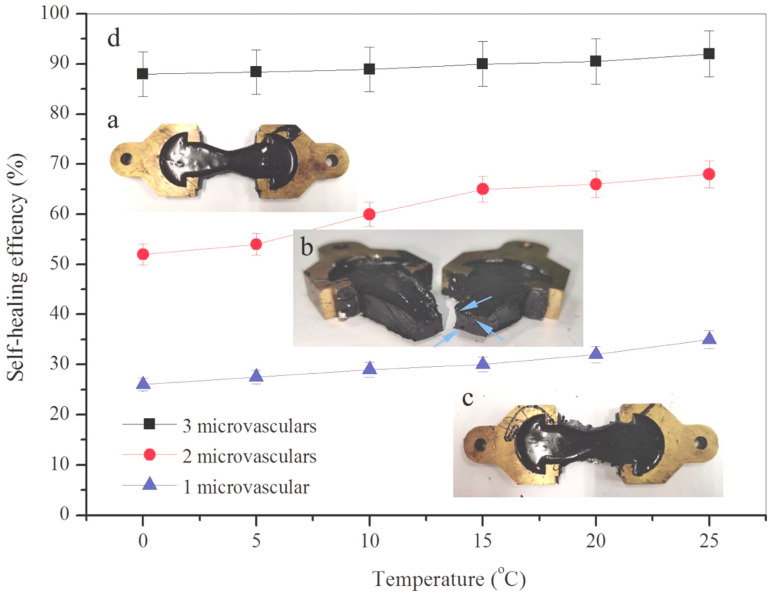
Self-healing efficiency (SHC) values of asphalt samples, (**a**–**c**) photos of self-healing process of bitumen, (**d**) SHC values of asphalt samples with 1–3 microvasculars influenced by self-healing temperature in one self-healing cycle under temperature of 0, 5, 10, 15, 20 and 25 °C.

Normally, self-healing phenomena are defined as a crack closure and strength recovery for a certain material. Asphalt is a temperature-sensitive engineering material, as bitumen has a visco-elastic nature. In other words, the strength recovery result of asphalt from the wetting and diffusion of bituminous molecules is usually attributed to the visco-elastic feature leading it to an acceleration of its mechanical properties. The above feature can be identified from the morphology of tension fracture. Figure 12a–c shows the photographs of asphalt samples with 1–3 microvasculars during the first self-healing cycle under temperatures of 5 °C. The asphalt sample deforms under this tension condition (Figure 12a). When the tension force reaches its breaking strength, the sample ruptures into two parts (Figure 12b). The arrows point to the fractured section of the three microvasculars. After the fracture interface is spliced, two parts are recombined. This sample was placed in the incubator to complete the first self-healing process under a temperature of 5 °C for 3 days (Figure 12c). Figure 12d shows the SHC values of asphalt samples with 1–3 microvasculars influenced by self-healing temperature in the first self-healing cycle, under temperatures of 0, 5, 10, 15, 20 and 25 °C, respectively. As asphalt is a temperature-sensitive material, a high temperature setting is not suitable for this tension test. From the trend of the curve, at the same temperature, more microvasculars can release more rejuvenator, and the asphalt’s self-healing efficiency is higher. This conclusion is consistent with the previous test results. The trend in data curves indicates that a higher temperature can enhance the SHC values for each asphalt sample, because higher energy can accelerate the molecule movement of bitumen and rejuvenator at the same time [13,26,27]. Under a temperature of zero, asphalt samples with 1–3 microvasculars have the SHC value of 26%, 53% and 88%. It needs to be emphasized that multi-self-healing will reduce the healing capability of asphalt samples. After a multiple repetitive self-healing under the same temperature, the molecules have reached a new equilibrium state. Without external energy, the self-healing will have an increased threshold.

### 3.8. Preliminary Analysis of a Mathematical Model of Self-Healing Efficiency

The self-healing capability of asphalt is normally defined as the recovery of mechanical properties and the disappearance of cracks [30]. Based on a physico-chemical theory, the self-healing process also can be considered as a molecule movement of bituminous material. Normally, penetration value and softening point can be used to identify the physical properties of bitumen. It has been determined that softer bitumen has a higher self-healing capability because of its higher penetration value and lower softening point [35]. Moreover, it is believed that the chemical structure of the aged bitumen has a great influence on its self-healing capability [27]. Bitumen can be regarded as a colloidal system consisting of high molecular weight asphaltene micelles dispersed or dissolved in a lower molecular weight oily medium [28]. With increases in service time, bituminous material will unavoidably lose its thixotropy due to the consumption of smaller molecules. Its flexibility will gradually decrease and cause significant losses in mechanical properties. At the same time, the viscosity of bitumen will increase because of the decrease in molecule movement. The above molecule movement can be enhanced with the help of rejuvenator. The addition of small molecules of rejuvenator will greatly reduce the hardness and aging of bituminous material and improve the ability of bituminous molecular movement. In this process, the release and diffusion play a role in determining the movement of rejuvenator.

**Figure 13:**
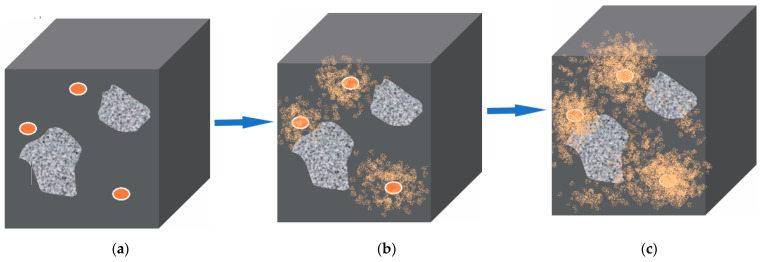
Illustration of the self-healing process of an asphalt sample with microvasculars containing rejuvenator, (**a**) rupture interface of an asphalt sample with broken microvasculars, (**b**) rejuvenator release out of microvasculars, and (**c**) rejuvenator diffusion continuously into asphalt under a certain temperature with time extension.

Figure 13 illustrates the mechanism of the self-healing process of an asphalt sample with multi-microvasculars containing rejuvenator. With the help of small molecules of rejuvenators, the rupture interface of asphalt can be closed more easily automatically [27]. When the asphalt sample has a crack or rupture, the embedded microvasculars at the same time are broken by the tip-stress of the crack, as shown in Figure 13a. Then, the oily rejuvenator is released out under the joint action of concentration difference dynamics and capillary dynamics [35]. Figure 13b shows the process of rejuvenator release and rapid penetration of rejuvenator in an asphalt sample. With time extension, rejuvenator can diffuse continuously into asphalt under certain temperatures (Figure 13c). The diffusion rate is affected by temperature, viscosity and aging degree of asphalt [36].

When the rejuvenator molecules diffuse into asphalt, the movement ability of bituminous molecules is also greatly enhanced at the same time [21]. Through entanglement and collusion of molecular segments, bituminous molecules can make cracks disappear quickly [37]. At a certain temperature, a larger area of rupture interface needs more time for molecular movement. That means that the self-healing capability cannot be enhanced rapidly. On the other hand, more rejuvenator appearance at the rupture interface can accelerate the movement capability of bituminous molecules. This accelerates the improvement of self-healing efficiency of materials. Based on the above analysis of the self-healing mechanism of asphalt using a microvascular containing rejuvenator, a conclusion can be drawn that these two main factors influence its self-healing efficiency (SHC). The SHC value is a function with two variables as shown in Equation (3):

(3) $$\mathrm{SHC}=f_{1}(A_{\mathrm{inter}f},m_{\mathrm{rejuv}})$$

where *A*_interf_ is the rupture interface area and amount of m_rejuv_ is the rejuvenator amount at the rupture interface. Meanwhile, data of m_rejuv_ is controlled by the temperature (*T*), broken number of microvasculars (*N*), viscosity of rejuvenator and rejuvenator time (*t*) as shown in Equation (4):

(4) $$m_{\mathrm{rejuv}}=f_{2}(N,T,t,\eta )$$

The diffusion behavior of rejuvenator molecule in asphalt is another factor to be considered in a self-healing process. It has been reported in previous work that the self-healing process of asphalt using microvasculars is a periodic continuous movement of liquid rejuvenator, including steps of penetration, release, capillary, and diffusion [15]. The diffusion coefficient (*D*) is defined as a ratio between the molar flux and the gradient of concentration. In this study, the microstructure of asphalt and temperature are considered as two main factors influencing the *D* values. It must be mentioned that the aged degrees of bitumen (*A*_d_) greatly influence the diffusion behaviors of rejuvenator [34]. In order to simplify the complexity, only one type of aged bituminous material (40/50) was selected as a diffusion matrix to test the basic diffusion phenomenon and rules in this work. The preliminary measurements will give a broad range of values, which greatly help us to understand the movement rules of rejuvenator molecules in bitumen. Usually, Fick’s law can be used to mathematically describe diffusion behaviors, and this simplified theory can describe many diffusion behaviors with less influence from chemical structures. However, the microvascular self-healing has been proven to have a more complex diffusion process [29]. Rejuvenator flows out and penetrates, and then diffuses into an aged bituminous material with a non-line rule. Therefore, the D values are controlled by both processes of penetration and diffusion. A preliminary mathematical model is in Equation (5):

(5) $$D=ae^{-e^{\left(b(T-T_{c})\right)}}$$

where *a*, *b*, and *c* are constants. Although this rule may not be an accurate calculation, it is still a guide to design the microstructure of microvasculars containing rejuvenator.

Considering the above self-healing mechanism, the self-healing process is determined by both steps of rejuvenator penetration and diffusion. In other words, the self-healing process is affected by the parameters of *A*_interf_, m_rejuv_, *D*, *T* and *t* at the same time as shown in Equation (6):

(6) $$\mathrm{SHC}=f_{3}(A_{\mathrm{inerf}},m_{\mathrm{rejuv}},D,T,t)$$

It is well known that temperature (*T*) is another important factor greatly influencing the SHC value. In previous work, it has been reported that the bituminous molecule movement depends on the time–temperature superposition principle [33]. Normally, the SHC model of asphalt can be described by using the time–temperature superposition principle, as shown in Equations (7) and (8) [33,34]:

(7) $$\mathrm{SHC}={\left[1+{\left(\frac{m}{t\cdot \alpha_{T}}\right)}^{\frac{\log2}{n}}\right]}^{\frac{n}{\log2}}\cdot 100$$

(8) $$\log\alpha_{T}(T)=\frac{\Delta E_{a}}{2.303R}\left(\frac{1}{T}-\frac{1}{T_{0}}\right)\;$$

where _αT_ is the time–temperature superposition shift factor, *m* and *n* are model parameters, Δ*E_a_* is the apparent activation energy (unit: J/mol) and R is the universal gas constant (8.314 J/(mol·K). Su [7] has investigated the time–temperature dependence of self-healing bitumen materials by using microcapsules that contained rejuvenator. SHC values show that an increase in temperature can enhance the self-healing effects of each healing cycle because a higher temperature can accelerate the molecule movement for the bitumen and rejuvenator molecules. Therefore, a higher energy can reduce the molecular motion resistance. This phenomenon has been explained in detail in previous work [15,17,27]. Healing has the lowest SHC value, which is consistent with the above conclusion. Su [33] also found that multi-self-healing reduced the healing capability of bituminous materials. This conclusion is consistent with a previous analysis that self-healing process has a threshold without an external energy. Bituminous molecules reached a new equilibrium state without an enhanced self-healing capability after a repeated healing process under a consistent temperature.

To sum up, we can give a function to describe the relationship between SHC and influencing factors as shown in Equation (9):

(9) $$\mathrm{SHC}=f_{4}(A_{\mathrm{inerf}},\varphi_{1}(N,T,t,\eta ),\varphi_{2}(D),\varphi_{3}\left(T,t\right))$$

where *A*_inerf_ is the area of rupture of interface, *ϕ*_1_ is a function of parameters of *N*, *T*, *t* and *η*, *ϕ*_2_ is a function of parameter of *D*, and *ϕ*_3_ is a function of parameters of *T* and *t*. Obviously, there are many factors influencing the self-healing mentioned above, and its functional relationship may be very complex. In spite of its complexity and uncertainty, the model is based on the self-healing structure and self-healing mechanism of asphalt using microvasculars, which guides SHC research. In the follow-up study, the above mathematical model will be further optimized and fitted, which is as close to the real self-healing process as possible, so as to guide the design of the material structure and the prediction of SHC more conveniently.

## 4. Conclusions

Self-healing function is one of the most basic characteristics of natural and self-healing materials, and is considered as a widely recognized research field in the 21st century. In recent years, the self-healing concept has been introduced into the field of asphalt pavement materials. In this work, the self-healing capability of asphalt/microvascular material had been investigated through the microstructure and its mechanical properties. The preliminary results allow the following to be concluded:

(1) Micro-CT and SEM were applied to observe the internal microstructure of asphalt with microvasculars. No interface separation was found between microvasculars and bitumen matrix. Moreover, no accumulation or tangles appeared for microvasculars dispersing in asphalt.
(2) The self-healing phenomenon of microcracks was proved with the help of diffusing out rejuvenator from the broken microvasculars. From the perspective of diffusion dynamics, rejuvenator molecules have a diffusion capability based on the motive force of a concentration gradient.
(3) A tensile test was designed to evaluate the self-healing efficiency of various asphalt samples considering the factors of microvasculars content, healing-time and healing-temperature. Self-healing efficiency was enhanced with more broken microvasculars in the rupture interface of an asphalt sample. During two self-healing cycles, the self-healing efficiency of the asphalt sample with three microvascular per 1 cm^2^ of a broken interface could reach 80% and 86%.
(4) The self-healing efficiency of the asphalt sample was increased over time over the same self-healing cycle. In this work, asphalt samples could nearly recover to more than 80% of tensile load value of its original state after 5 days of self-healing. On the other hand, self-healing efficiency values showed that an increase in temperature could enhance the self-healing effects for each asphalt sample because higher temperatures could accelerate the molecule movement for the bitumen and rejuvenator molecules. Therefore, a higher energy can reduce the molecular motion resistance.
(5) A preliminary mathematical model was also deduced that the self-healing efficiency was determined based on the self-healing steps including rejuvenator release, penetration and diffusion. A function with many parameters is derived through the simplification of material structure. It will guide future numerical simulation analysis and practical application.

## Data Availability

Not applicable.
